# Hyperviscosity Syndrome in Immunoglobulin G4 (IgG4)-Related Disease and the Use of Therapeutic Plasma Exchange: A Case Report

**DOI:** 10.7759/cureus.70972

**Published:** 2024-10-07

**Authors:** Lennart Marahrens, Rhys Thomas, Tamir Malley

**Affiliations:** 1 Rheumatology, Royal Free London NHS Foundation Trust, London, GBR

**Keywords:** hypergammaglobulinaemia, hyperviscosity syndrome, igg4-related disease, polyclonal hyperviscosity, therapeutic plasma exchange

## Abstract

Immunoglobulin G4 (IgG4)-related disease is a rare multi-system immune-mediated inflammatory condition. Pathologically, it is associated with an increase in plasma IgG4 levels and tissue infiltration of IgG4-secreting plasma cells of any organ. Clinical features vary, but it usually presents with the diffuse enlargement and dysfunction of the affected organ. IgG4-related disease leading to hyperviscosity syndrome from polyclonal hypergammaglobulinaemia has only been described in two case reports, and no consensus regarding the treatment of this organ- and life-threatening complication exists. We report the case of a 50-year-old man who was admitted with symptoms of hyperviscosity and an extremely elevated IgG4 level. A presumptive diagnosis of IgG4-related disease causing hyperviscosity syndrome was made. He underwent a therapeutic plasma exchange with a resolution of his symptoms. In view of the likely diagnosis of IgG4-related disease and only refractory improvements in IgG4 levels, immunosuppression with steroids and rituximab was commenced. This led to a sustained fall in IgG4 levels and remission. Hyperviscosity syndrome should be recognised as a complication of IgG4-related disease, and therapeutic plasma exchange can be employed in the emergency treatment of this life-threatening condition.

## Introduction

In this case study, we report on a patient with hyperviscosity syndrome (HVS) and polyclonal hypergammaglobulinaemia secondary to immunoglobulin G4-related disease (IgG4-RD). IgG4-RD is an immune-mediated inflammatory condition characterised by tissue infiltration of IgG4-secreting plasma cells which can affect virtually any organ [1]. Raised IgG4 levels are often associated with IgG4-RD, but HVS due to an increase in plasma viscosity secondary to very high IgG4 levels appears to be an extremely rare complication, which to our knowledge has only been described twice previously. We discuss the utility and limitations of therapeutic plasma exchange in the emergency treatment of HVS secondary to IgG4-RD prior to the establishment of a definitive diagnosis and initiation of effective immunosuppression.

## Case presentation

A 50-year-old Ethiopian man presented to our hospital with a six-month history of pruritus, weight loss (~20 kg), and fatigue. His past medical history was significant for ulcerative colitis with subtotal colectomy, oesophageal stricture, hypothyroidism, and a diagnosis of primary sclerosing cholangitis, which had been made clinically and radiologically in the absence of tissue biopsy. Investigations showed a conjugated hyperbilirubinaemia with obstructive liver biochemistry, hyponatraemia with normal serum osmolality, and eosinophilia. Blood test results are summarised in Table 1. Protein electrophoresis revealed a polyclonal increase in gamma globulins without paraproteinaemia. His IgG was significantly elevated at 47.7 g/L (normal range: 7-16), and an IgG subclass analysis was not performed. Eosinophilia screen (strongyloides, schistosomiasis, filaria serology; anti-neutrophil antibodies, tryptase) and interferon-gamma release assay were negative. HIV antibodies, anti-nuclear antibody testing, and rheumatoid factor were negative, with no clinical features of Sjögren's syndrome. He was treated for suspected pneumonia with antibiotics and was discharged with further outpatient follow-up by infectious diseases, hepatology, and rheumatology.

**Table 1:** Summary of laboratory investigations Improvement of laboratory abnormalities associated with IgG4-related disease over time in response to treatment. IgG4: immunoglobulin G4; IgG: immunoglobulin G

Laboratory values	Presentation with pruritus and weight loss	Presentation with hyperviscosity syndrome	After the first plasma exchange	After the second plasma exchange	Prior to starting steroids and rituximab	Follow-up 24 days after the first rituximab
Haemoglobin (135-170 g/L)	103	78	85	84	86	136
White blood cells (3.5-11.0 × 10^9^/L)	8.17	9.50	6.97	9.49	5.26	8.29
Eosinophils (0-0.5 × 10^9^/L)	1.35	1.7	1.33	2.30	0.83	0.05
Bilirubin (0-21 μmmol/L)	61	58	44	29	43	10
IgG (7-16 g/L)	47.7	72	37.6	27.3	44.4	15.1
IgG4 (0.04-0.86 g/L)	-	40.48	-	-	-	5.17
Plasma viscosity (1.4-1.75 mPa)	3.21	>7	2.40	1.95	-	-

**Figure 1:**
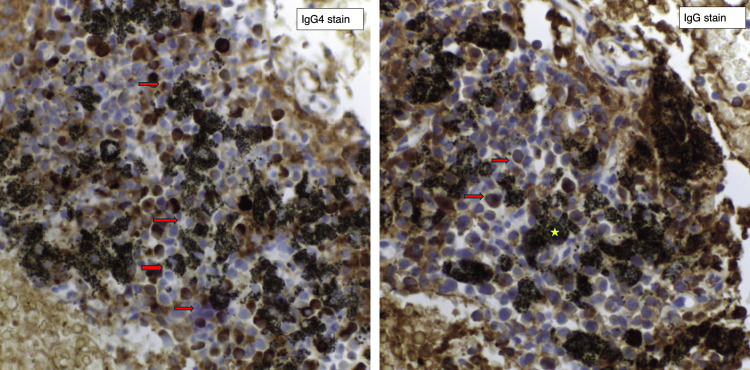
IgG4 and IgG stains at 40× magnification Red arrow: IgG4 and IgG staining cells of the respective stains. Yellow star: anthracosis within the lymphoid tissue. IgG4: immunoglobulin G4; IgG: immunoglobulin G

One month following discharge, the patient re-presented with worsening pruritus, a new non-specific headache, and blurred vision. Ophthalmological examination revealed venous tortuosity and blot haemorrhages. Polyclonal serum IgG had increased further to 72 g/L, and plasma viscosity was unrecordably high (>7 mPa, normal range: 1.40-1.75). In view of the apparent HVS, he underwent two therapeutic plasma exchanges (3L exchange, 2L 4.5% human albumin solution (HAS) and 1L Hartmann's solution) with the resolution of headache and vision changes and corresponding initial fall in IgG4 and plasma viscosity. Three days after the plasma exchange, IgG levels started to rise again; however, the patient remained asymptomatic. Results of IgG subclass analysis sent prior to plasma exchange returned a grossly elevated IgG4 of 40.8 g/L, with other IgG subclasses within the normal range. A fluorodeoxyglucose-positron emission tomography/computed tomography (FDG-PET/CT) scan demonstrated small volume low-grade metabolically active lymph nodes above and below the diaphragm with no definitive malignant lesions or infection identified. Histology of an upper paratracheal lymph node obtained during the admission (endobronchial ultrasound (EBUS) station 2R) showed an increase of IgG4-expressing cells compared to IgG cells (Figure 1). However, due to the poor quality of the obtained tissue, a specific ratio could not be given. Importantly, no evidence of malignancy was seen in that sample. TB-polymerase chain reaction (TB-PCR), acid-fast bacillus (AFB), and extended culture were negative. No other biopsies were taken in the absence of an amenable target.

**Figure 2:**
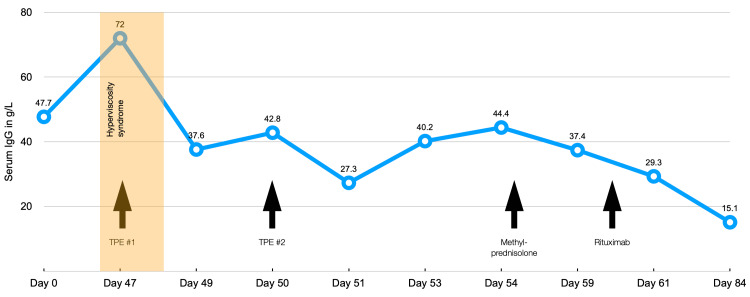
IgG levels over time in response to therapeutic plasma exchange, steroids, and rituximab Occurrence of symptoms of hyperviscosity shown in yellow. Plasma exchange only led to a temporary decrease in IgG levels, albeit enough to improve the patient's symptoms of hyperviscosity. A sustained fall in IgG was seen following immunosuppression with steroids and rituximab. IgG: immunoglobulin G

The diagnosis of IgG4-RD was made based on the very high IgG4 levels (>30× upper limit of normal), supportive biopsy findings, and the clinically compatible syndrome. The patient was commenced on steroids and rituximab with a sustained improvement in his symptoms and fall in IgG, IgG4, and plasma viscosity (Figure 2). He remained asymptomatic at follow-up one month after treatment initiation.

## Discussion

HVS

HVS comprises a constellation of clinical signs and symptoms caused by an increase in whole-blood and/or plasma viscosity [2]. Depending on the underlying aetiology, these classically include mucosal bleeding (epistaxis, gastrointestinal), neurological deficits (seizures, stroke mimics, headaches), and visual disturbances (retinal haemorrhage, papilloedema, tortuous vessels). Presentations can be vague and often vary significantly between patients, frequently leading to delayed diagnosis. In our patient, plasma viscosity was significantly elevated, leading to the characteristic signs (retinal haemorrhage and vein tortuosity) and symptoms (headaches, blurry vision) of HVS.

HVS can be caused by an abnormal elevation of any cellular (erythrocytes, platelets, or leukocytes) or acellular components (immunoglobulins) of the blood [3]. Among the latter, HVS is commonly associated with a monoclonal increase in immunoglobulins, most frequently IgM, such as that seen in Waldenstrom macroglobulinaemia or multiple myeloma [4]. Therapeutic plasma exchange has been established as the standard of care for patients presenting acutely with symptomatic hyperviscosity [5]. Case reports of polyclonal increases in immunoglobulins have been described in infections (e.g., HIV, hepatitis) and autoimmune diseases (e.g., Sjögren's syndrome, rheumatoid arthritis) [6]; however, these are rare and no clear treatment guidelines exist. The admission of our patient out of hours was complicated by the fact that no local guidelines or experience existed regarding the initiation of plasma exchange for patients with polyclonal HVS.

The evidence behind the use of plasma exchange in hypergammaglobulinaemias has so far largely been based on monoclonal gammopathies, particularly those affecting IgM. Among the different immunoglobulins, IgM is much more likely to cause HVS than the other isomers due to its larger molecular size, pentameric structure, and tendency to form immune complexes [7]. Fortunately, plasma exchange is particularly suitable for the removal of IgM. The majority (~80-90%) of IgM can be found in the intravascular space and is therefore more readily removed by plasma exchange, leading to a rapid decline in plasma viscosity. In contrast, being a smaller and more diffusible isomer, a substantial proportion of IgG (~50%) is found in the extravascular space, making removal by plasma exchange less effective [8]. This was also seen in our patient, who, after initial improvement in IgG levels after plasma exchange, showed a rebound increase in IgG, albeit whilst remaining asymptomatic. From a practical perspective, plasma exchange in hyperviscosity due to IgG can furthermore be associated with more significant fluid shifts due to the higher extravascular concentration of IgG, resulting in expert guidelines to recommend the use of additional crystalloid or colloid during plasma exchange in these patients [5]. In our patient, plasma exchange was done using 3L exchange, with 2L 4.5% HAS and 1L Hartmann's solution, and he remained stable throughout the transfusion.

IgG4-RD

A disease which is prototypical for an increase in polyclonal IgG is that of IgG4-RD. IgG4-RD is an immune-mediated inflammatory condition characterised by tissue infiltration of IgG4-secreting plasma cells which can affect virtually any organ [1]. IgG4-RD is rare, with an estimated prevalence of five per 100,000 persons [9]. It is an under-diagnosed entity, and previously described diseases such as autoimmune pancreatitis and retroperitoneal fibrosis are now recognised as being part of the spectrum in which IgG4-RD can present. It is not confined to patients of a particular age, sex, or ethnicity. Often, IgG4-RD presents indolently, the cardinal feature being masses in or the diffuse enlargement of the involved organ. It has a propensity to affect the salivary glands, orbits and lacrimal glands, pancreas and biliary tract, and retroperitoneum. Vascular involvement (peri-aortitis and aortitis) and thyroiditis (Riedel's thyroiditis) also occur, and weight loss and lymphadenopathy are common [10]. The patient discussed in this case report had a history of primary sclerosing cholangitis, oesophageal stricture, and hypothyroidism, which were made at a different institution. It is possible that these have been manifestations of long-standing IgG4-RD. The observed hyperbilirubinaemia, which had previously been presumed to be secondary to primary sclerosing cholangitis, entirely resolved following the initiation of steroids and rituximab. Steroids and rituximab have no role in the treatment of primary sclerosing cholangitis and would have been unlikely to significantly affect bilirubin levels [11], but would be expected to lead to an improvement in IgG4-related sclerosing cholangitis [12].

Diagnosis of IgG4-RD is frequently suspected because of incidental findings on cross-sectional imaging or histopathology. Laboratory evaluation includes serum IgG4, which is often elevated but is neither necessary nor sufficient to establish a diagnosis of IgG4-RD. Using a high cut-off of 27 g/L has yielded a specificity of 91% for IgG4-RD in one study [13]. The patient reported in this study presented with an IgG4 of 40.8 g/L, over 30× the upper limit of normal. Other abnormalities are hypocomplementaemia and a raised IgE, both seen in this patient. Findings on biopsy include storiform fibrosis, obliterative phlebitis, and tissue infiltration of mainly IgG4-positive plasma cells [1]. Ideally, a biopsy should be obtained from one of the affected organs with the exception of lymph nodes due to lower specificity and sensitivity [14]. As with many rheumatological diseases, there are no universally agreed-upon diagnostic criteria for IgG4-RD.

The mainstay of initial therapy to induce remission are steroids, and the vast majority of patients show a clinical response with prednisolone monotherapy. Lack of steroid responsiveness should prompt consideration of an alternate diagnosis. In patients with multi-organ disease or very high IgG4 concentrations, combination therapy with rituximab has shown beneficial outcomes and has also shown some promise as a steroid-sparing monotherapy. Azathioprine and mycophenolate have been used as second-line options [15].

Hyperviscosity in IgG4-RD

Elevated serum IgG4 levels as seen in IgG4-RD result in polyclonal hypergammaglobulinaemia. As described above, IgG is only rarely associated with hyperviscosity, and as such very few cases of IgG4-RD causing HVS have been reported. Wong and colleagues [16] describe a 41-year-old male patient who developed HVS eight years after having shown first manifestations of IgG4-RD. At the time of his initial presentation with lacrimal swelling, gamma globulin was normal (IgG and its subclasses were not measured). Seven years later, he presented with pneumonia and marked hypergammaglobulinaemia but no clinical symptoms or signs of hyperviscosity. Shortly after this, he re-presented with visual blurring and retinal haemorrhages. IgG and IgG4 levels were unmeasurably high, and he was thus diagnosed with HVS. A single run of plasma exchange initially led to a fall in his plasma viscosity and clinical improvement. This, however, proved short-lived, and one week later, his vision deteriorated again. He required serial plasma exchanges every 3-4 weeks to maintain IgG levels <40 g/L, leading to the resolution of his visual symptoms. A diagnosis of IgG4-RD was only made nine months following this after the patient presented again, this time with large vessel vasculitis. He was commenced on rituximab and fludarabine (a purine analogue) to induce and maintain remission.

Chen and colleagues [17] report a second patient with HVS secondary to IgG4-RD. This 21-year-old female was initially diagnosed with HVS secondary to hypereosinophilic syndrome, a diagnosis which was later revised to IgG4-RD [18]. She underwent plasma exchange with good clinical response following three cycles and was able to maintain remission on prednisolone and azathioprine. What stands out from both these patients is their relatively young age at the time of diagnosis, 41 and 21, respectively. The average age of diagnosis of IgG4-RD had previously been estimated to be around 60 [19]. In a small case series, Chen and colleagues [18] describe (in addition to the two patients above) a further five previously published reports of polyclonal hyperviscosity which they suspect may have been due to IgG4-RD, albeit none of these had a definitive diagnosis made. Four out of these five patients were treated with plasma exchange. All required maintenance immunosuppressive therapy, and interestingly, out of their total seven patients, only three had a good response to steroids. The average age of these seven patients at the time of diagnosis was 41.2 years, almost 20 years younger than what one would expect from previous studies on IgG4-RD. Their relatively young age and their variable response to steroids could suggest that HVS mainly occurs in patients with particularly aggressive forms of IgG4-RD. What is not surprising is the fact that plasma exchange alone is insufficient to induce or maintain remission in patients with HVS from IgG4-RD. As discussed previously, plasma exchange is less suited to clear IgG than other immunoglobulins due to its high concentration in extravascular compartments. Furthermore, plasma exchange is unlikely to affect the ongoing production of polyclonal IgG4 as seen in IgG4-RD. As such, a rebound in hyperviscosity and high IgG4 levels are to be expected. As described above, this was demonstrated in the patient presented in this case report, whose IgG levels and plasma viscosity only showed a sustained decrease following the initiation of steroids and rituximab. 

## Conclusions

This case report of HVS in IgG4-RD, which adds to the two previously documented cases, demonstrates three points. Firstly, HVS should be recognised as a complication of IgG4-RD. Whilst it is likely to be an uncommon manifestation, early recognition and treatment are important to prevent this organ- and life-threatening disease. Secondly, HVS may be a marker of disease severity and possibly a predictor of lack of steroid response. This may therefore be an argument to support an earlier initiation of steroid-sparing agents, such as rituximab in this case study, in order to induce remission more reliably. Thirdly, plasma exchange, a therapy normally utilised in monoclonal hypergammaglobulinaemias, has its value in the acute management of polyclonal hyperviscosity seen in IgG4-RD. Due to the unique properties of IgG compared to IgM, several sessions of plasma exchange may be required, and improvement and plasma hyperviscosity and symptoms are likely to be only temporary. Nevertheless, it can act as a holding measure to prevent severe organ dysfunction prior to the initiation of a more definitive immunosuppressive treatment. Guidelines should therefore incorporate the use of plasma exchange in the acute setting of HVS secondary to IgG4-RD.

## References

[1] Kamisawa T, Zen Y, Pillai S, Stone JH (2015). IgG4-related disease. Lancet.

[2] Fahey JL, Barth WF, Solomon A (1965). Serum hyperviscosity syndrome. JAMA.

[3] Kwaan HC, Bongu A (1999). The hyperviscosity syndromes. Semin Thromb Hemost.

[4] Mehta J, Singhal S (2003). Hyperviscosity syndrome in plasma cell dyscrasias. Semin Thromb Hemost.

[5] Connelly‐Smith L, Alquist CR, Aqui NA (2023). Guidelines on the use of therapeutic apheresis in clinical practice - evidence-based approach from the Writing Committee of the American Society for Apheresis: the ninth special issue. J Clin Apher.

[6] Zhao EJ, Cheng CV, Mattman A, Chen LY (2021). Polyclonal hypergammaglobulinaemia: assessment, clinical interpretation, and management. Lancet Haematol.

[7] Stone MJ (2009). Waldenström's macroglobulinemia: hyperviscosity syndrome and cryoglobulinemia. Clin Lymphoma Myeloma.

[8] Cervantes CE, Bloch EM, Sperati CJ (2023). Therapeutic plasma exchange: core curriculum 2023. Am J Kidney Dis.

[9] Wallace ZS, Miles G, Smolkina E (2023). Incidence, prevalence and mortality of IgG4-related disease in the USA: a claims-based analysis of commercially insured adults. Ann Rheum Dis.

[10] Khosroshahi A, Stone JH (2011). A clinical overview of IgG4-related systemic disease. Curr Opin Rheumatol.

[11] Angulo P, Batts KP, Jorgensen RA, LaRusso NA, Lindor KD (2000). Oral budesonide in the treatment of primary sclerosing cholangitis. Am J Gastroenterol.

[12] Manickavasagan HR, Wood R, Kindsfather S, Munoz SJ (2016). Dramatic effect of rituximab in immunoglobulin g4-related primary sclerosing cholangitis. Am J Gastroenterol.

[13] Carruthers MN, Khosroshahi A, Augustin T, Deshpande V, Stone JH (2015). The diagnostic utility of serum IgG4 concentrations in IgG4-related disease. Ann Rheum Dis.

[14] Sato Y, Kojima M, Takata K (2009). Systemic IgG4-related lymphadenopathy: a clinical and pathologic comparison to multicentric Castleman's disease. Mod Pathol.

[15] Zhang W, Stone JH (2019). Management of IgG4-related disease. Lancet Rheumatol.

[16] Wong PC, Fung AT, Gerrie AS (2013). IgG4-related disease with hypergammaglobulinemic hyperviscosity and retinopathy. Eur J Haematol.

[17] Chen LY, Lai EJ, Collins DR, Ostrow DN, Sreenivasan GM (2010). A young woman with episodic angioedema, papilledema, and eosinophilia. Am J Hematol.

[18] Chen LY, Wong PC, Noda S, Collins DR, Sreenivasan GM, Coupland RC (2015). Polyclonal hyperviscosity syndrome in IgG4-related disease and associated conditions. Clin Case Rep.

[19] Brito-Zerón P, Ramos-Casals M, Bosch X, Stone JH (2014). The clinical spectrum of IgG4-related disease. Autoimmun Rev.

